# Impact of COVID-19 infection on the dialysis population prospective, observational, nationwide study

**DOI:** 10.1007/s11255-022-03368-1

**Published:** 2022-09-22

**Authors:** Ali AlSahow, Ahmed AlQallaf, Anas AlYousef, Hamad Bahbahani, Yousif Bahbahani, Bassam AlHelal, Heba AlRajab, Abdullah AlMuhaiteeb, Heba Shalaby, Mohamed Elabbadi, Mohammad Elsebaei, Emad Abdallah, Medhat Ayoub, Aissar AbouTrabeh, Maryam AlSarrajji, Abdullah AlAwadhi, Rajeev Kumar

**Affiliations:** 1 Division of Nephrology, Jahra Hospital, Jahra, Kuwait; 2 Division of Nephrology, Jaber Hospital, AlSalam, Kuwait; 3 Division of Nephrology, Amiri Hospital, Kuwait City, Kuwait; 4 Division of Nephrology, Mubarak Hospital, Jabriya, Kuwait; 5 Division of Nephrology, Adan Hospital, Hadiya, Kuwait; 6 Division of Nephrology, Farwaniya Hospital, Sabah AlNasser, Kuwait; 7 BRA IRCH, AIIMS, Delhi, India

**Keywords:** COVID-19, Dialysis, Hemodialysis, Mortality, Kuwait

## Abstract

**Introduction:**

Hemodialysis (HD) patients are at increased risk of severe COVID-19 infection but infection rates vary. Our objectives are to describe COVID-19 positive HD patients’ characteristics, infection rates, and factors associated with mortality in HD COVID-19 cases in Kuwait.

**Methods:**

Data on demographics, comorbidities, and treatments received, as well as mortality for HD patients admitted to hospitals for COVID-19, from 1/March to 31/July 2020, prospectively collected and analyzed.

**Results:**

A total of 141 infected HD patients were admitted (Mean age 58 ± 16.1; Males 56%), representing 7% of the total HD population and 0.2% of all COVID-19 cases during the study period. Of those 141 infected HD patients, 27 (19%) died, and this represents 6% of total COVID-19-related mortality and 27% of the total HD mortality. In contrast, total covid-19-related mortality of all positive cases was only 0.7%, and total HD mortality during the study period was only 5%. COVID-19-positive HD patients who died were older and 59% were males. However, the differences were not statistically significant. Of the 61 infected HD patients who needed to be switched to continuous kidney replacement therapy (CKRT), 34% died, and of the 29 infected HD patients who needed admission to intensive care, 65% died.

**Conclusion:**

HD population represents a small fraction of the total population; however, positive HD COVID-19 cases represent a sizable proportion of COVID-19 cases and a significant percentage of total COVID-19-related mortality, and total HD mortality.

## Introduction

A novel coronavirus leading to severe respiratory infection (coronavirus disease 2019, COVID-19) was first identified in Wuhan, China in December 2019 [1]. It was identified as the seventh coronavirus that could infect humans and was named severe acute respiratory syndrome—Coronavirus 2 (SARS-CoV-2) [2]. COVID-19 infection was declared a pandemic by the World Health Organization (WHO) due to its high rate of transmission, on 11 March 2020 [3]. As of the end of November 2021, more than 260 million people had been infected and more than 5,200,000 died worldwide [4]. WHO have defined severe disease as “a patient with severe acute respiratory illness (fever and at least one sign/symptom of respiratory disease, e.g., cough, shortness of breath; AND requiring hospitalization)” [5]. One report from the United States showed that about 70% of patients with comorbidities infected with COVID-19 required hospital admission and more than 90% of deaths were in patients with underlying conditions [6]. Multiple reports from different countries listed some risk factors for severe COVID-19 infection that include advanced age and comorbidities, such as diabetes mellitus (DM), cardiovascular disease (CVD), and hypertension (HTN) [7, 8]. Data from the Global Burden of Disease (GBD) collaboration, using 2017 prevalence data and UN population estimates for 2020, have estimated the global population at high risk for severe COVID-19 to be 1.7 billion people, comprising 22% of the global population, of whom 349 million would require hospital admission if infected. This study found that CKD was the most prevalent risk factor for severe COVID-19 worldwide [9].

Hemodialysis (HD) patients are at higher risk of infections including COVID-19 due to compromised immune status from uremia, metabolic derangement, inflammation, oxidative stress, and intestinal dysbiosis [7, 10]. Moreover, HD patients have to visit the dialysis center for HD thrice weekly, which means they cannot adhere to the stay-home policy and cannot avoid close contact with other people [7]. The first report from China on COVID-19 and HD showed that 42 patients out of 230 were infected, and 13 of the 42 died (5.65% of the total HD population) [11]. Results from the European renal association – European dialysis and transplantation association (ERA-EDTA) registry earlier in the pandemic, between Feb 1st to April 30th, 2020, indicate a high mortality due to COVID-19 in dialysis patients reaching 21% [12]. Another report from US facilities showed a 25% COVID-19 mortality rate where older age, heart disease, and markers of frailty were highly associated with mortality [13]. To prevent the spread of infection in HD centers, the nephrology community in Kuwait has implemented the recommendations published in international guidelines [14–17]. The objectives of this multi-center prospective study are to describe the status of COVID-19 infection in HD patients, the characteristics of such patients, and factors associated with increased mortality in HD covid-19 patients in Kuwait.

## Methods

In the first wave of COVID-19 infection in Kuwait, from March 1, 2020, to July 31, 2020, the Ministry of Health (MoH) policy was to admit all COVID-19 positive cases to public hospitals, regardless of the severity of the infection, and treat for free. MoH HD centers serve 95% of the HD population, and 100% of the peritoneal dialysis (PD) population. The remaining 5% of the HD population receive HD in the private sector. Such patients were transferred to and treated in MoH hospitals in the event of contracting COVID-19 infection. Asymptomatic patients were tested based on a history of contact with positive cases. Patients were discharged only when they were asymptomatic and PCR negative for SARS-CoV-2 as per MoH policy. Once a dialysis patient is admitted to a hospital due to COVID-19 infection, we started to collect data on demographics and comorbidities. Patients then were followed while inpatient prospectively for up to four weeks (or less if discharged or died earlier) to record treatments received and other data in case of death. All HD patients above the age of 18 admitted to MoH hospitals with COVID-19 infection were enrolled. We had no exclusion criteria, and we had no refusal to consent. Data were collected from six general MoH hospitals with nephrology service that includes HD treatment. The MoH prospectively collected COVID-19 infection rates and mortality rates in the country and reported results daily. We compared our results to the rates reported by MoH for the entire population. We also looked at the percentage of COVID-19-related mortality to the total mortality in the HD population during the study period. We also looked at mortality rates at 6 months post-infection for those who survived the acute event. The study protocol was approved by the Joint Ministry of Health and the Kuwait University Committee on Medical Research, and all recruited patients consented to study entry before starting data collection.

The data analysis was performed using the statistical software Stata16 (StataCorp, College Station, TX, USA). The data were summarized using descriptive statistics in the form of mean/SD for continuous variables and number/percentage for categorical variables. The 95% CI was obtained using the exact method. Chi-square test was performed to compare the mortality in covid-19 and non-covid-19 HD patients. The univariable binary logistic regression with a robust estimator was applied to find the association of patient characteristics with mortality in Covid-19 positive patients on HD. The *p* < 0.2 in univariable logistic regression was included in the multivariable logistic model with a robust estimator. A *p* value less than 0.05 was considered as significant.

## Results

### Total COVID-19 cases in the general and the dialysis population

The total number of COVID-19 positive cases confirmed by PCR from a nasopharyngeal swab from March 1, 2020, to July 31, 2020, was 66,957 [18]. (less than 1.5% of the total population of 4,600,000 or 146 per 10,000 of population) [19]. The total number of HD patients infected with COVID-19 was 141 (7% of the total HD population in 2000 and 0.2% of the total number of confirmed COVID-19 cases) (Table 1).

**Table 1:** Baseline characteristics and lines of management of all COVID-19 positive dialysis patients:

Patients’ characteristics	HD
Total HD population in Kuwait at time of study	2000
Total COVID-19 positive cases (% of total corresponding dialysis population)	141 (7%)
Male/female (%)	79 (56%)/62 (44%)
Mean age (male/female)	58 ± 16.1 (54.0 ± 17.5/62.0 ± 13.2)
Comorbidities (%)	
DM	94 (66.6%)
HTN	131 (93%)
Cardiac disease (CAD/CHF)	69 (49%)
Respiratory diseases (Asthma/COPD)	23 (16%)
Therapies receive by all cases	
Corticosteroids	30 (21.3)
Hydroxychloroquine	7 (4.96)
Remdesivir	0 (0.0)
Tocilizumab	2 (1.42)
Lopinavir/ritonavir	7 (4.96)
Convalescent plasma	1 (0.71)
ICU admission	29 (20.6%)
Intubation/assisted ventilation	23 (16%)

*HD* Hemodialysis, *PD*  Peritoneal Dialysis, *DM* Diabetes Mellitus, *HTN* Hypertension, *CAD* Coronary Artery Disease, *CHF* Congestive Heart Failure, *COPD* Chronic Obstructive Airway Disease, *ICU* Intensive Care Unit

The mean age for HD patients with COVID-19 was 58 ± 16.1 (males 54.0 ± 17.5 were significantly younger than females 62.0 ± 13.2; *p* = 0.002). HTN, DM, heart disease, and chronic respiratory illness were present in 93%, 67%, 49%, 16%, respectively.

Steroids were given to 21% of patients, and hydroxychloroquine to 5%. Intensive care unit (ICU) admission was required for 20.6% of the HD patients, with 80% of those requiring mechanical ventilation (Table 1). Conventional HD was switched to continuous kidney replacement therapy (CKRT) in 61 patients (34%). The number of admitted patients who were on CKRT exceeds the number of patients admitted to ICU because many hemodynamically stable patients in the general medical wards were put on CKRT due to a lack of access to dialysate water in the inpatient wards in many MoH hospitals.

### Outcomes

The total number of deaths due to COVID-19 infection in Kuwait during the study period was 447 (0.67% of the total number of positive cases) [18]. The total mortality in the HD population during that period regardless of the cause was 100 (5% of the total HD population), however, mortality due to COVID-19 infection in HD patients was 27% of the total mortality in the HD population, 6% of the total COVID-19 related mortality in the country, and 19% of the total HD COVID-19 positive cases. The mortality was significantly higher in covid-19 HD patients than in non-covid-19 patients 19.1% vs 3.9% OR = 5.80 [95% CI 3.58–9.37; *p* < 0.001].

HD Patients who died from COVID-19 were older with a mean age of 62.9 ± 12.1 vs 56.9 ± 15.2 for HD patients who survived but the mean difference between them was not statistically significant *p* = 0.058. HD patients who died also had respiratory illness more frequently (25% vs 14%) and 59% were males. Of the 61 patients who were switched to CKRT, 34% died. Of the 29 HD patients admitted to ICU, 65% died. Of those who died, 95% were on assisted ventilation, and 63% received corticosteroids. Except for age, no other factor was significantly associated with mortality in covid-19 HD patients in univariable logistic regression with a robust variance estimator. Linearity of age in the logit scale was checked by adding a square term in the model (Table 2). Multivariable logistic regression considered the factors that had *p* < 0.2 in univariable analysis with a robust estimator, effect of age also became insignificant between the survivors and non-survivors (Table 2).

**Table 2:** Risk factors for mortality in hemodialysis patients infected with COVID-19:

Variable		Survivor (*n* = 114)	Non-survivor (*n* = 27)	*p* value	Odds ratio [95% CI]	Adjusted odds ratio [95% CI]
Age (years)	Mean (SD)	56.93 ± 15.23	62.91 ± 12.14	0.041	1.03 [1.00–1.07]	1.03 [0.995–1.055]; *p* = 0.094
Gender—male	*N* (%)	63 (55.3)	16 (59.3)	0.708	1.18 [0.50–2.77]	–
DM	*N* (%)	74 (64.9)	20 (74.1)	0.368	1.54 [0.60–3.96]	–
HTN	*N* (%)	104 (91.2)	27 (100.0)	0.209	5.47 [0.68–718.39]*	–
Cardiac diseases	*N* (%)	55 (48.2)	18 (66.7)	0.090	2.14 [0.89–5.18]	1.63 [0.66–4.04]; *p* = 0.287
Respiratory illnesses	*N* (%)	17 (14.9)	7 (25.9)	0.178	2.00 [0.73–5.45]	1.79 [0.67–4.78]; *p* = 0.246

*DM* Diabetes Mellitus, *HTN* Hypertension

^*^Using Firth logistic regression applied due zero count

A 6-months follow-up on HD patients who survived COVID-19 revealed that 16.7% of the 114-survivor died, five remained Oxygen dependent, and the rest recovered completely.

There were an additional seven PD patients who contracted the virus during the same period, representing 3% of the total PD population and 4.8% of the dialysis patients infected with SARS-CoV-2. Although the total number of COVID-19 positive PD patients was low, mortality in COVID-19 positive PD cases approached 43%, much higher than the mortality rate in COVID-19 HD patients (19%). PD Patients who died from the infection were older with a mean age of 62.6 vs 49.5 for survivors.

## Discussion

Reported SARS-CoV-2 infection and mortality rates in the HD population are variable, [8, 9, 12, 13]. partly because early reports failed to adequately assess chronic kidney disease (CKD) prevalence and its impact on COVID-19 disease severity and instead focused on CKD-associated conditions (CVD, HTN, and DM) [20]. Reports were also influenced by sample sizes and the use of in-hospital populations groups of severely ill patients with an estimated risk that may not be generalizable to the broader kidney replacement therapy (KRT) population [12]. However, CKD and especially dialysis, is a common and strong risk factor for both infection and mortality [8, 9]. Studies from China, Europe, and North America reported higher mortality and more severe disease in HD patients compared to the general population [21–29].

COVID-19 infection rates in the dialysis population varied from 2.2 [22] to 19.6%, [25] with our study reporting a rate of 7%, and mortality rates varied from 16.2 [30] to 52%, [31] while our rate was 19%. Both rates are much higher than the general population in these reports and in our cohort. Dialysis patients also had a shorter time from symptom onset to ICU admission compared to other groups [32].

Our study confirms findings in other reports that infection and mortality rates in HD patients are higher than in the general population. Important risk factors for mortality in dialysis patients include older age and male gender [12, 13]. Although non-survivors in our HD population were older, mainly male, and had more comorbidities than survivors, differences were not statistically significant.

There is a paucity in the medical literature when it comes to COVID-19 infection and chronic PD patients. European data showed that PD patients represented 4% of total dialysis patients infected with COVID-19 (31 of 768 patients) and 5% of the total mortality in that group (29% of the infected PD group) [33]. A paper analyzing a single center experience reported that out of the total hospitalized dialysis population with the infection, 2.6% were PD patients (11 patients), with an 18% mortality rate in the PD group infected with the virus [34]. Another paper, again analyzing a single center experience reported a 3.4% PD share (2 patients) with no deaths [27]. A Canadian study reported a 1.8% infection rate in the entire in-center HD population for the province of Ontario versus 0.8% in the PD population [35]. PD patients represented 4.8% of the total infected dialysis patients, and 3% of the total PD population, and had a much higher death rate (~ 43%).

With regard to pharmacological treatment of COVID-19; corticosteroids, remdesivir, and tocilizumab have clinical benefits, but hydroxychloroquine and lopinavir-ritonavir combination are ineffective [36]. Steroids were used in only 21% of our HD patients, and the other medications were used in an even much lower proportion. Steroids were thought to be effective in cases with severe COVID-19-associated pneumonia followed by tocilizumab which was an off-label use at that time, but its use was restricted to ICU patients who met certain criteria as per the MoH policy at that time. Therefore, it is hard to draw any conclusion on the effect of such therapies on HD patients with COVID-19.

Unfortunately, the medical literature on the issue of the long-term impact of COVID-19 infection in the dialysis population was scarce. This study shed light on the impact of COVID-19 in dialysis patients with results that clearly show the associated increased morbidity and mortality. Such data is crucial to help policymakers and directors of hemodialysis centers in formulating more effective strategies to protect vulnerable patients with comorbidities and reduce pressure on health care systems.

### Strengths and limitations

Our study aimed for complete national coverage, eliminating sampling bias found in smaller and non–population-based studies. However, lack of testing of positive cases (lack of symptoms, refusal of testing, or not reporting to the dialysis center), may have led to an overestimation of mortality, albeit small. Identifying at-risk populations is important for the design of effective interventions that aim to reduce the risk of transmission to vulnerable patients like in the dialysis population. Since we do not have complete clinical data for all the HD patients thus comparison was not done between the COVID-19 HD and non-COVID-19 HD patients.

## Conclusion

Despite the fact the HD population represents only a small fraction of the total population, they represent a sizable proportion of the total COVID-19 positive cases and a significant percentage of the total COVID-19 related mortality. This study highlights the increased susceptibility of the HD population to COVID-19 infection, which is associated with a high rate of mortality in Kuwait, although lower than mortality rates reported in Europe and the United States. It also highlights the importance of adherence to screening, prevention, and isolation policies to reduce the spread of infection and save lives. It also lends support to the calls worldwide to give dialysis patients priority to receive the COVID-19 vaccine.

## Data Availability

Data can be provided by the corresponding author in case of a reasonable request. This is not a clinical trial.
